# Optimizing Adaptive Therapy Based on the Reachability to Tumor Resistant Subpopulation

**DOI:** 10.3390/cancers13215262

**Published:** 2021-10-20

**Authors:** Jiali Wang, Yixuan Zhang, Xiaoquan Liu, Haochen Liu

**Affiliations:** 1 School of Pharmacy, China Pharmaceutical University, Nanjing 210009, China; 3119010072@stu.cpu.edu.cn (J.W.); yxzhang@stu.cpu.edu.cn (Y.Z.); 2 Center of Drug Metabolism and Pharmacokinetics, China Pharmaceutical University, Nanjing 210009, China

**Keywords:** adaptive therapy, reachability-based adaptive therapy, restore index, intra-competition, osimertinib

## Abstract

**Simple Summary:**

The intra-competition among tumor subpopulations is a promising target to modify and control the outgrowth of the resistant subpopulation. Adaptive therapy lives up to this principle well, but the gain of tumors with an aggressive resistant subpopulation is not superior to maximum tolerated dose therapy (MTD). How to integrate these two therapies to maximize the outcome? According to the model and system reachability, the ‘restore index’ is proposed to evaluate the timing of the transition from the treatment cycle of adaptive therapy to high-frequency administration, and to juggle the benefits of intra-competition and killing of the sensitive subpopulation. Based on the simulation and animal experiment, the effectiveness of this method in treating tumors with an aggressive resistant subpopulation has been confirmed.

**Abstract:**

Adaptive therapy exploits the self-organization of tumor cells to delay the outgrowth of resistant subpopulations successfully. When the tumor has aggressive resistant subpopulations, the outcome of adaptive therapy was not superior to maximum tolerated dose therapy (MTD). To explore methods to improve the adaptive therapy’s performance of this case, the tumor system was constructed by osimertinib-sensitive and resistant cell lines and illustrated by the Lotka-Volterra model in this study. Restore index proposed to assess the system reachability can predict the duration of each treatment cycle. Then the threshold of the restore index was estimated to evaluate the timing of interrupting the treatment cycle and switching to high-frequency administration. The introduced reachability-based adaptive therapy and classic adaptive therapy were compared through simulation and animal experiments. The results suggested that reachability-based adaptive therapy showed advantages when the tumor has an aggressive resistant subpopulation. This study provides a feasible method for evaluating whether to continue the adaptive therapy treatment cycle or switch to high-frequency administration. This method improves the gain of adaptive therapy by taking into account the benefits of tumor intra-competition and the tumor control of killing sensitive subpopulation.

## 1. Introduction

Adaptive therapy (classic adaptive therapy) [1,2,3,4,5,6] is developed from tumor intra-competition to delay the outgrowth of the resistant subpopulation. It has been well explored in animal experiments and clinical trials, especially in prostate cancer and breast cancer [2,3,4]. The prerequisites for classic adaptive therapy to surpass MTD are clear, especially in the study of Strobl MAR et al. [7]. That is, strong tumor intra-competition, slow-growing resistant subpopulation, initial low resistant subpopulation, or high turnover tumor cell. These prerequisites ensure the competitive advantage of sensitive subpopulations in the tumor system. However, this means when the tumor has an aggressive resistant subpopulation, the performance of adaptive therapy is not superior to MTD [7]. An aggressive resistant subpopulation refers to the resistant subpopulation that proliferates rapidly or has a strong competitive ability or high carrying capacity or high initial content. Because in this case, the outcome of MTD is not worse than that of adaptive therapy, indicating that it is worth exploring to integrate the MTD’s principle of killing as many tumor cells as possible into classical adaptive therapy to delay the occurrence of end events.

The classic adaptive therapy regimen consists of repeated treatment cycles, the duration of the treatment cycle generally includes a period of administration and withdrawal [2,3,5]. The indicator of administration and withdrawal is the level of tumor volume or PSA relative to the beginning of adaptive therapy [2,3,5]. This treatment cycle maintains the intensity of tumor intra-competition and appropriate tumor burden. Compared with MTD, classic adaptive therapy takes advantage of the tumor intra-competition to inhibit the proliferation of resistant subpopulations, while MTD mainly benefits from the killing of sensitive subpopulations by high-frequency administration to control tumor burden. However, as the treatment cycle of classic adaptive therapy continues, the proportion of resistant subpopulation gradually increases, and the duration of the treatment cycle continues to decrease until the end event is reached. This is the general end of classic adaptive therapy [5], which means that the impact of intra-competition is reduced at the later period of classic adaptive therapy.

Hence, it is meaningless to maintain intra-competition in the later period of classic adaptive therapy. At this time, killing a large number of sensitive cells like MTD may increase the outcome of classic adaptive therapy. To maximize the outcome of competitive stress and killing of the sensitive subpopulation, the timing to bridge these two therapies should be chosen wisely. Therefore, the control degree of competitive stress on resistant subpopulations should be quantitatively evaluated. In this way, the timing of treatment switching from the treatment cycle of classic adaptive therapy to high-frequency administration of MTD can be explored effectively. Then system reachability would be introduced to assess the degree of control. In this tumor system, system reachability mainly relies on the proportion of sensitive subpopulation, because it is easily regulated by drugs, while the resistant subpopulation can only be indirectly regulated by sensitive subpopulation. Therefore, an indicator of reachability should be constructed that is associated with the two participants and can predict the duration of each treatment cycle. Based on the purpose of integrating classic adaptive therapy and MTD, the threshold of the reachability indicator would be explored in the study to evaluate the timing of treatment switching, and the feasibility of this reachability-based adaptive therapy would also be verified.

In this research, the fitness cost for the resistant subpopulation of tumor system was avoided, because this property does not always exist in resistant subpopulations, unless the resistant mechanism is mediated by energy-consuming methods, such as multidrug resistance transporters [8,9]. Then this study was conducted in EGFR-mutant non-small cell lung cancer (NSCLC). The main resistant mechanism of tyrosine kinase inhibitor (TKI) is the absence of sensitive mutations, so the fitness cost can be avoided. Here we focused on a common TKI resistant mechanism, namely the emergence of KRAS mutation subpopulation [10]. Therefore, the tumor system was constructed by EGFR-mutant sensitive and KRAS-mutant resistant cell lines, and the modulation drug was osimertinib. To improve the poor performance of classic adaptive therapy in the above cases, this study explored the implementation of reachability-based adaptive therapy. The Lotka-Volterra model was introduced to describe the tumor system quantitatively. Combined with the model, an indicator related to system reachability would be proposed to evaluate the duration of each treatment cycle and the timing of switching to the high-frequency administration. Furthermore, the applicability of the indicator and reachability-based adaptive therapy would be verified.

## 2. Methods

### 2.1. Cell Culture

Human lung adenocarcinoma cells (H1975 and A549) were purchased from the National Collection of Authenticated Cell Cultures (Shanghai, China) and confirmed to be mycoplasma-free by Gmyc-PCR kit (Yeasen, Shanghai, China, cat# 40601ES10). All cell lines were cultured in RPMI-1640 (Gibco (Thermo Fisher Scientific), Waltham, MA, USA, cat# 21870076) containing 10% fetal bovine serum (Gibco, cat# 10099141C) and 1% Penicillin-Streptomycin (5000 U/mL) (Gibco, cat# 15070063).

In terms of drug sensitivity, H1975 is sensitive to osimertinib, and A549 is resistant to osimertinib. In addition, H1975 contains EGFR T790M mutation, A549 contains KRAS G12S mutation. H1975/GFP (green fluorescent protein) cells, expressing GFP to track cells, were transfected by eGFP-puromycin resistant lentivirus (Genomeditech, Shanghai, China, cat# GM1002) and selected with 0.5 mg/L puromycin to maintain the expression of label proteins. A549/RFP (mCherry fluorescent protein)/luc (firefly luciferase) cells, expressing RFP and firefly luciferase, were transfected by RFP-Luciferase-puromycin resistant lentivirus (Genomeditech, cat# GM-10619LV) and selected with 1.6 mg/L puromycin (MedChemExpress, Shanghai, China, cat#HY-B1743A) to maintain the expression of label proteins.

### 2.2. Nutrition Restriction Model Establishment and Growth Curve Acquisition

The nutrition restriction model was used to demonstrate one aspect of the competition between subpopulations in vitro. To obtain the growth curve, 24-well plates containing 1:1 mixed H1975/GFP and A549/RFP/luc, the corresponding single cell line as control groups were detected under high-content imaging analysis system Opera Phenix (PerkinElmer, Waltham, MA, USA) from 24 h after cell planting. The number of cells was calculated by the Image Analysis and Evaluation module of high-content analysis software Harmony (version 5.0, PerkinElmer) every 12 h [11]. As for nutrition restriction, the culture medium was diluted by adding an equal volume of PBS (Gibco, cat#20012027, pH 7.2) directly, and the culture media was replaced after the first time point of imaging. To avoid serious pH deviation and nutrient consumption, the diluted medium was replaced every 12 h.

### 2.3. Animal

Animal experiments were carried out in compliance with the Chinese law on animal welfare. All animal care and experimental procedures were approved by the animal ethics committee of the Pharmaceutical Animal Experiment Center of China Pharmaceutical University. 5 to 7-week-old female BALB/c nude mice (Charles River, Beijing, China) were allowed to acclimatize (in groups of 3–4 mice per cage) for at least 7 days prior to the experiment and were maintained on a 12-h light:12-h dark cycle with free access to standard rodent chow and water.

### 2.4. Animal Experiment of Model Fitting

In this experiment, mice were inoculated with different cells to obtain the data for fitting. The cell suspension was inoculated subcutaneously (~5 × 10^6^ cells per mouse) into the left forelimb flank of mice. Firstly, mice were inoculated with H1975 or A549 to fit the tumor growth curve. Then, mice inoculated with H1975 or A549 would be given osimertinib 20 mg/kg/day to fit the parameters of the drug effect. After that, H1975 and A549 were mixed at a ratio of 9:1 and then inoculated to get the competition parameters. The content of H1975 and A549 in the mixed tumor was obtained by In vivo imaging every 3–4 days. Tumor volume was measured by caliper two to three times a week, and calculated according to the formula $V=\pi \times \left(\mathrm{lengh}\times width^{2}\right)/6$. For the administration of osimertinib (MedChemExpress, cat# HY-15772), mice were given intragastric osimertinib (20 mg/kg) in normal saline (3% Tween 80). The vehicle control group in the drug effect experiment was given the corresponding solvent without the drug.

### 2.5. Animal Experiment of AT2 Validation

To compare various therapy protocols, mice were inoculated subcutaneously with mixed tumor cells (~5 × 10^6^ cells per mouse), the mixed ratio of H1975 and A549 was 9:1 before inoculation. Because the sensitive subpopulation should occupy a high proportion at the beginning of therapy, then several rounds of treatment cycles can be carried out. When tumor volume reached 220–300 mm^3^, mice were randomly grouped according to tumor volume and weight. Tumor volume was measured by caliper two to three times a week and the A549 content in the mixed tumor was collected sparsely. As for the content of H1975, because the duration of in vivo imaging of H1975 for each mouse was more than half an hour, for animal welfare considerations and to ensure the physical condition of the animal, the content of H1975 was not collected at this stage.

### 2.6. In Vivo Imaging

When tumor volume was about 200 mm^3^, IVIS Spectrum (PerkinElmer) was used to evaluate the content of each subpopulation of the mixed tumor through in vivo imaging, and the data was processed using Living Image software (version 4.3.2, PerkinElmer). Serially diluted H1975/GFP or A549/RFP/luc cells were cultured in a 96-well black plate with a transparent bottom. This was used to build a quantification database of bioluminescence and fluorescence intensity with cell numbers. And for luminescence imaging, D-Luciferin potassium salt (Beyotime, Shanghai, China, cat#ST196) should be given to mice 12 min in advance by intraperitoneal injection at a dose of 150 mg/kg body weight. After that, fluorescence imaging tomography or diffuse luminescence imaging tomography was acquired to reconstruct a fluorescent or luminescence source in 3D space and calculate the absolute intensity of that source at depth [12,13], then we can get the cell number of the mixed tumors. As for the conversion from cell number to subpopulation volume, using the normal growth data of mixed tumors, the cell number of two subpopulations and the corresponding tumor volume were regressed to get the cell volumes of H1975 and A549. Therefore, based on the reconstruction results of tomography and corresponding quantification database, Living image software can calculate the cell number of H1975 and A549, then the cell number can be converted to subpopulation volume according to the cell volume.

### 2.7. Model Establishment

Lotka-Volterra model was used to describe the competition of subpopulations [14]. The tumor cell turnover was included in the intrinsic growth rate and was not considered as a separate item. The efficacy of osimertinib mainly contributed to the death of H1975, and we assumed that its efficacy was positively correlated with an intrinsic growth rate of H1975. Considering the individual variation and the distribution of population parameters, the nonlinear mixed-effects model was selected as an appropriate statistical framework [15]. This method can reflect the overall changes of the samples, and consider the differences between individuals. The observation described in the model is shown in Equation [1], which can be divided into structural model f and residual error model g. The structural model f describes the variation of observations to the variables. Residual errors are the difference between model predictions and observations, and the residual error model is set as a proportional error model. Here, i represented individual and j represented corresponding time point. We assumed the set of biological related individual parameters $\phi_{i}$ in this model follows a log-normal distribution, like the distribution of $\phi_{i}$ in equation [1], vector μ indicates the fixed effects, and ω indicates the variance of individual random effects. Moreover, the fixed effect is the mean of the population parameters under the pre-assumed distribution and the random effect means the variation of the population parameters between individuals. $t_{ij}\;$ are the time points, the residual errors $\varepsilon_{ij}$ follows standard normal distribution, and ξ is the regression parameter in the residual error model.

(1) $$y_{ij}=f\left(\phi_{i},t_{ij}\right)+g\left(\phi_{i},t_{ij},\xi \right)\varepsilon_{ij}\;\phi_{1},\ldots ,\;\phi_{I}\sim LN\left(\mu ,\omega \right)$$

The detailed model of normal growth condition was as following equations [2,3], the model of tumor given osimertinib was as following equation [4]. *H* and *A* represents the content of H1975 and A549, $C_{OSI}$ represents the concentration of osimertinib, $E_{OSI}$ represents the efficacy of osimertinib on H1975. The biological related parameters, intrinsic growth rate, r, carrying capacity, K, competition parameters, h and a were considered interindividual variability, and the subscript (h,a) of parameter indicated that it belonged to H1975, A549, respectively. The estimation of these biologically related parameters included fixed effects and random effects.

(2) $$\frac{dH}{dt}=r_{h}\times H\times \left(1-\frac{H+a\times A}{K_{h}}\right)$$

(3) $$\frac{dA}{dt}=r_{a}\times A\times \left(1-\frac{A+h\times H}{K_{a}}\right)\;$$

(4) $$\frac{dH}{dt}=r_{h}\times H\times \left(1-\frac{H+a\times A}{K_{h}}\right)-C_{OSI}\times E_{OSI}\times H\times \left(1-\frac{A}{A+H}\right)$$

### 2.8. Parameter Estimation

The above model-related parameters were estimated by Monolix 2019R2 (Lixoft SAS, Antony, France, 2019). The fitting results of parameters contained typical value and inter-individual variability. The typical value was the mean of the population parameter and the inter-individual variability was the variance of the parameter distribution. The optimization method of parameters was the maximization of the likelihood function to obtain the appropriate values by stochastic approximation expectation-maximization algorithm [16]. The model was evaluated based on the goodness-of-fit and individual-weighted residuals (IWRES) to detect misspecifications in the structural and residual error models. And the η-shrinkage was calculated to analyze the degree shrinkage of the individual parameter to the center of the population distribution, which was used to judge the reliability of diagnostic plots [15,17].

### 2.9. Simulation of Therapies

#### 2.9.1. Part1 Estimation Individual Parameters of Experiment Samples

Before the start of the first treatment cycle of classic adaptive therapy and reachability-based adaptive therapy, the observations of tumor volume and single point of A549 content were collected. Based on this data, the individual estimates were obtained from individual empirical Bayes estimates assessed by fitting to the population model.

#### 2.9.2. Part2 Estimation the Appropriate Threshold of Restore Index for Reachability-Based Adaptive Therapy

In reachability-based adaptive therapy, there was an indicator of system reachability for evaluating whether to interrupt the treatment cycle. The process of finding the threshold of this interruption point was divided into two steps, the first step was to find the range of the interruption point, and the second step was to find the appropriate value.

The first step was to get the treatment plans of reachability-based adaptive therapy that were not inferior to the classic adaptive therapy through simulation. The regimen of reference classic adaptive therapy was shown in the following Treatment protocols. The tumor composition at the beginning of the simulation was the average value of the mixed tumor samples of the animal experiment of model fitting, and the biological related parameters were the typical value of parameters representing the characteristics of the experimental animals. In the simulation of reachability-based adaptive therapy, the start time of the first treatment cycle was set to different time points, and the treatment cycle was set to 3 times. In each treatment cycle, the duration of drug administration and withdrawal was set at different lengths of time. In each treatment cycle, we can skip the drug withdrawal part and continue to administer it to achieve the purpose of interrupting the treatment cycle in advance. The above simulation treatment plans with the same or better outcomes as the reference classic adaptive therapy were screened out to obtain the range of the threshold.

Then, each integer in the above range was used as a threshold to simulate the outcomes of reachability-based adaptive therapy under different conditions. The different condition means the intrinsic growth rate of resistant subpopulation was defined as 50–150% relative to the typical value of H1975, carrying capacity was defined as 80–120% relative to the typical value of H1975, and competition parameter was defined as 0–200% relative to the typical value of H1975. Finally, the value that maximizes the performance improvement of tumors with aggressive resistant subpopulation and minimizes the similarity to classical adaptive therapy was selected as the threshold.

#### 2.9.3. Part3 Model Validation of Adaptive Therapies in Animal Experiment

Combined with the administration of each sample in the experiment, GUN Octave (version 6) simulated the changes of tumor volume and A549 content of each sample over time with individual parameters.

### 2.10. Treatment Protocols

The starting time point of each therapy was calculated from the tumor volume reaching 220–300 mm^3^, the end event was set as tumor volume reaching 1000 mm^3^ [2]. In addition, the duration of osimertinib administration was at least two days to reduce the uncertainty of the drug effect of a one-day administration. The general regimen for each therapy in experiment and simulation is as follows:

Control: Normal saline with 3% Tween 80 was given after grouping.

Positive control: Osimertinib was given 20 mg/kg/day when tumor volume was as close as possible to 1000 mm^3^.

MTD: Osimertinib was given 20 mg/kg/day after grouping. The MTD is aimed at eradicating all cancer cells [18]. Moreover, MTD cures tumors on the assumption that resistance subpopulation appears after treatment. Therefore, in this study, osimertinib was administered at the maximum dose as soon as possible.

Classic adaptive therapy (AT1): To maximize the use of tumor intra-competition, the first treatment cycle of adaptive therapy started from the tumor volume as close as possible to 1000 mm^3^. Then the treatment cycle consisted of two days of osimertinib administration and drug withdrawal until the tumor volume was as close as possible to 1000 mm^3^. If the tumor volume does not decrease with two days of osimertinib administration, osimertinib would not be withdrawn.

Reachability-based adaptive therapy (AT2): The start of the treatment cycle was the same as AT1. However, if the tumor volume reduction was less than 100 mm^3^ with two days osimertinib administration, the treatment cycle would be interrupted and osimertinib would not be withdrawn. As for the reason for 100 mm^3^, it was explained in the results.

### 2.11. Statistical Analysis

In all figures, the data presented were representative of at least 3 independent experiments. Data were expressed as the mean ± SEM and statistically analyzed using the R (3.6.3) and SPSS (version 21.0). The significance analysis of growth curve among different groups used multivariate analysis of variance followed by a post-hoc Bonferroni test, drug effect, and effect of the conditioned medium used analysis of *t*-test every time point by SPSS and visualized by ggplot2. And survival analysis of different treatments was used pairwise comparisons between group levels with corrections by the survminer package. The acceptable level of significance set at *p* < 0.05; *p*-values were shown in figures as *: *p* < 0.05, **: *p* < 0.01 and ***: *p* < 0.005, ****: *p* < 0.0001, N.S.: not significant.

## 3. Results

### 3.1. Cell Competition in Restriction of Space or Nutrition

**Figure 1:**
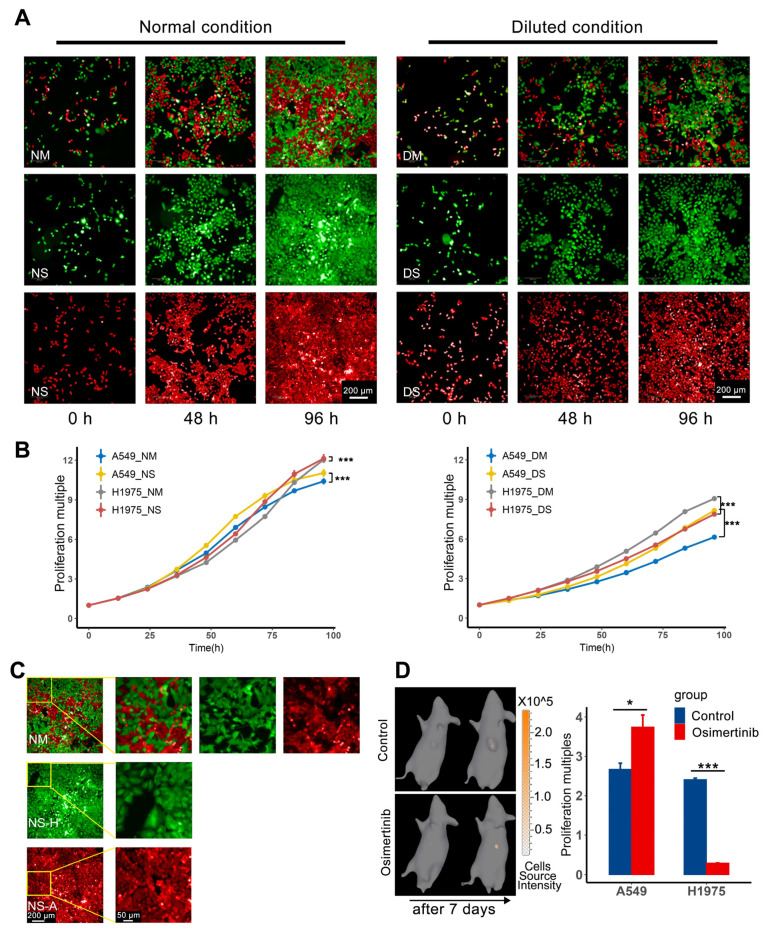
Cell competition under resource constraints. (**A**) Observe the proliferation of H1975/GFP and A549/RFP separately and mixed culture in different media (green H1975 and red A549) at 0, 48, 96 h. (**B**) The growth curves of the two cell lines in different media. The proliferation multiple was defined as the number of cells at each time point for subpopulation at each time point divided by the number of cells at the initial time. Multivariate analysis of variance followed by a post-hoc Bonferroni test was performed between single and mixed cells in the same medium, the results showed that mixed culture would have a significant effect on cell proliferation both in normal and diluted medium (*n* = 4 per group; *p*-value for all tests < 0.005, *** *p* < 0.005.). (**C**) The detailed graphs at the end time point were used to compare the cell morphology in single and mixed conditions. The first row is the case of mixed culture under normal conditions, the second line is the case of H1975 culture under normal conditions alone, and the third line is the case of A549 culture under normal conditions alone. (**D**) The left picture is the 3D-bioluminescence measurement of A549, the upper row is the control group, the lower row is the osimertinib group (the raised shadow is the protruding tumor). The histogram on the right is the proliferation multiples of A549 and H1975 in two groups. The proliferation multiple was defined as the final volume of a subpopulation divided by its initial volume. The two subpopulations are significantly different between the two groups (*n* = 3 per group; paired t-test, A549 in control group vs. osimertinib group *p*-value = 0.04016, H1975 in control group vs. osimertinib group *p*-value = 0.00025. * *p* < 0.05, *** *p* < 0.005.).

The competition between subpopulations mostly occurs under resource-constrained conditions, such as space and nutrition constraints [19,20,21]. Here, the competition between H1975/GFP and A549/RFP was tested in the above two aspects. The overall growth performance of H1975/GFP and A549/RFP separately and mixed culture at 0, 48, 96 h was shown in (Figure 1A), and details of each time point were in Supplementary Figure S1. The corresponding growth curve was shown in (Figure 1B). The left panel showed the cells in a normal culture medium with obvious space constraints in the later stage, and the right panel showed the condition of the cells in a diluted culture medium. The proliferation multiple was defined as the number of cells for subpopulation at each time point divided by the number of cells at the initial time. Multivariate analysis of variance followed by a post-hoc Bonferroni test showed that mixed culture would affect the growth of the two cell lines under space or nutrition constraints. Moreover, the effect of mixing two cell lines was stronger in the case of diluted culture than in the case of limited space.

The difference between the growth curves of the H1975 and A549 normal single (NS) group and dilution single (DS) group indicated that H1975 was more susceptible to nutrition restriction than A549, and A549 was more susceptible to space restriction. The influence of space restriction was mainly reflected in the end part of the growth curves of H1975_NS and A549_NS. When the two cell lines were mixed at a ratio of 1:1 under a normal culture medium, H1975 still maintained the advantage of obtaining space resources. (Figure 1C) showed the details of two cell lines at the end time point of the normal culture. Compared with the NS group, H1975 had more obvious deformation than A549 in the normal mixed (NM) group. This deformation variation maintained the same cell proliferation multiple of H1975 at the end of the NM and NS groups but resulted in a decrease in the proliferation multiple of A549 at the end of the NM group. In addition, the performance of the diluted mixed (DM) group was opposed to the original performance of the DS group. The proliferation of H1975 in the DM group was higher than that in the DS group. In contrast, this phenomenon was the opposite in A549. In other words, under the mixed and limited nutrition conditions, H1975 can maintain proliferation better than A549. In general, the difference in apparent proliferation rates of the two cell lines under different conditions indicated the result of competition under resource-constrained conditions, which constituted the basis of adaptive therapy.

### 3.2. In Vivo Cell Competition can Control the Growth of Resistant Subpopulation A549

In vitro experiments confirmed that these two cell lines compete for space and nutrition when resources are limited. And tumor cell lacks space and nutrition in vivo exactly [22,23,24,25]. Furthermore, the competition between sensitive subpopulation H1975 and resistant subpopulation A549 was confirmed in vivo. When the tumor volume exceeds 200 mm^3^, the composition of each tumor subpopulation of mixed tumor-bearing nude mice was evaluated, and the subpopulation volume was converted from the number of cells according to Supplementary Table S1. Following the results of cell composition and tumor volume, the mice were paired and randomly divided into the control group and osimertinib group. Mice in the osimertinib group were given osimertinib 20 mg/kg/day by gavage for 7 days. Then the tumor components were reassessed, the in vivo imaging result of A549 for one sample was shown on the left side of (Figure 1D). The subplot on the right side of (Figure 1D) was the proliferation multiple of two subpopulations, defined as the volume of A549 and H1975 divided by their initial volume in the control and osimertinib groups. The in vivo imaging of H1975 of the same sample was shown in Supplementary Figure S2. The volume of tumor subpopulation before and after the experiment was recorded in Supplementary Table S2. The results showed that after 7-days osimertinib administration, H1975 was killed massively, and the proliferation multiple of A549 increased significantly. Therefore, when the competitive inhibition of H1975 was lifted, the apparent proliferation rate of A549 in mixed tumors could be accelerated. This conclusion indicated that the reachability of the tumor system can be achieved by adjusting the content of H1975.

### 3.3. Model Parameter Estimates and Model Evaluation

The established model was shown in Equations [2,3,4]. The experiment data used for parameter estimation was the mice inoculated H1975 or A549 alone, which were randomly administered osimertinib (20 mg/kg/day) or vehicle. The effect of osimertinib on H1975 was shown in Supplementary Figure S3A. The comparison of the growth of A549 in control and osimertinib groups indicated that osimertinib had no obvious inhibitory effect on A549 as shown in Supplementary Figure S3B. To obtain the parameters of the competition between these two subpopulations, the experiment data was also obtained from mice inoculated with mixed tumor (H1975:A549 = 9:1) and randomly administered osimertinib (20 mg/kg/day) or vehicle. The model parameters were estimated by Monolix and listed in Supplementary Table S3.

**Figure 2:**
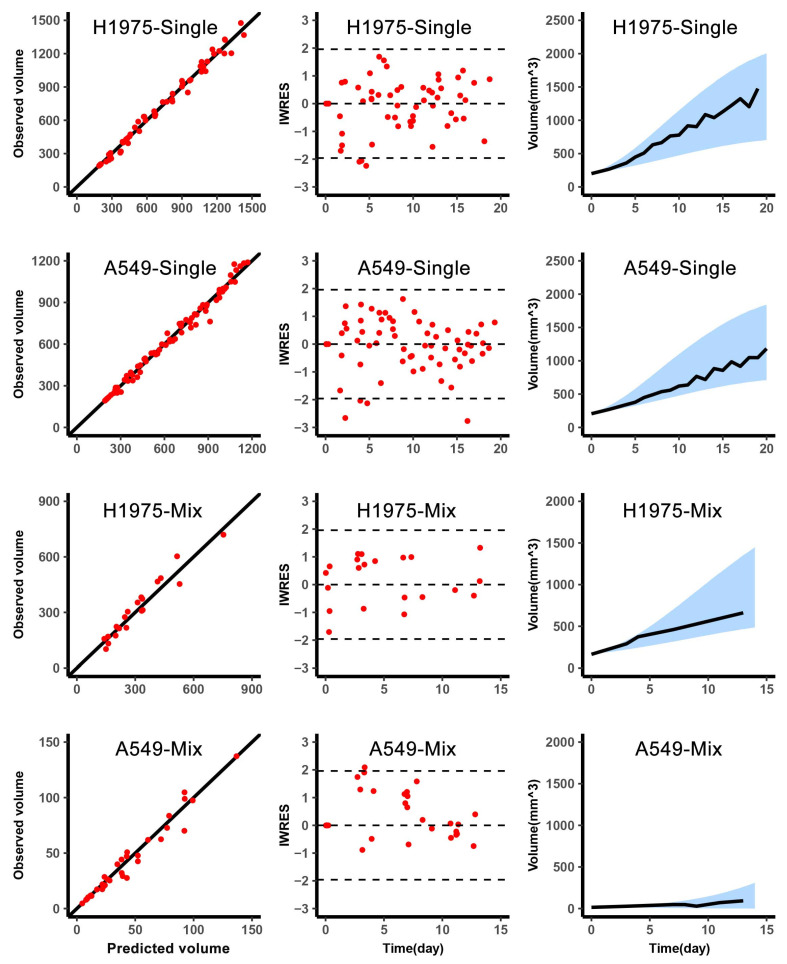
Graphical diagnostics of a single and mixed growth of H1975 and A549 growth in vivo. Observations versus individual predictions for H1975 in single (top, *n* = 6), A549 in single (second row, *n* = 7), H1975 in mixed (third row, *n* = 10), A549 in mixed (bottom, *n* = 10). Each row includes lines of identity (**left**), individual-weighted residuals (IWRES) versus time (**middle**), and the predicted volume versus time (**right**). In the subplots of predicted volume versus time, the blue shaded area represents the 90% confidence interval of the simulated median value. The line represents the median of the observations.

**Figure 3:**
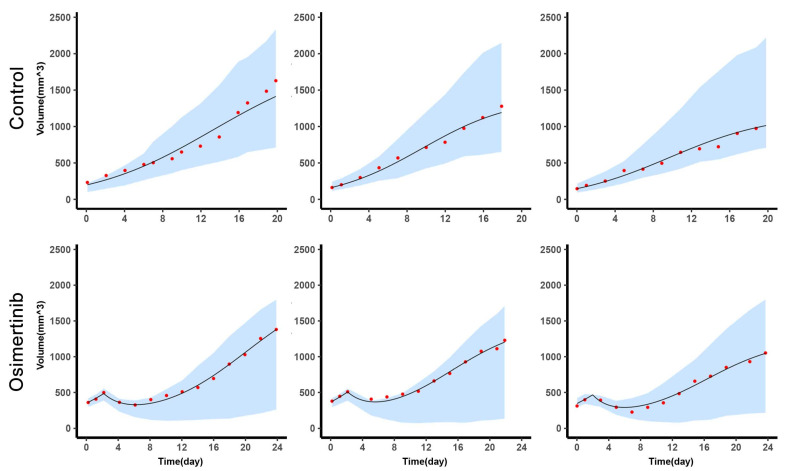
The efficacy of osimertinib in mixed tumors. The tumor volume change versus time for 3 samples in each group (top, control group, *n* = 10; bottom, osimertinib administration group, *n* = 3). In each subplot, the red dot represents the observed volume of mixed tumor, the line represents the individual prediction by simulation using the individual empirical Bayes estimates, the blue shaded area represents the 90% confidence interval of individual prediction.

The model described the volume dynamics of H1975 and A549 in single and mixed conditions well. The η-shrinkage of each individual parameter was within 30%, which confirmed the reliability of diagnostic plots [15,17]. As shown in the left column of (Figure 2), the predicted volume was closely related to the observed volume, the dots were located around the diagonal. The middle column was the IWRES versus time, and the distribution of IWRES was close to the standard normal distribution. Since the levels of H1975 and A549 were obtained by in vivo imaging every 3–4 days, the number of data points for H1975 and A549 in the mixed tumor was less than a single condition. Moreover, in the subplots of the right column, the median of the observed value was within the 90% confidence interval, which was based on the simulation using the median initial volume. (Figure 3) depicted the observed mixed tumor volume and the 90% confidence interval of predictions using individual parameters in control and osimertinib administration groups. As shown in (Figure 3), the apparent growth rate of the control group was faster than that of the osimertinib administration group. This suggested that the intra-subpopulation competition was more intense than the inter-subpopulation competition.

### 3.4. Schematic Diagram and Comparison of MTD, AT1, AT2

**Figure 4:**
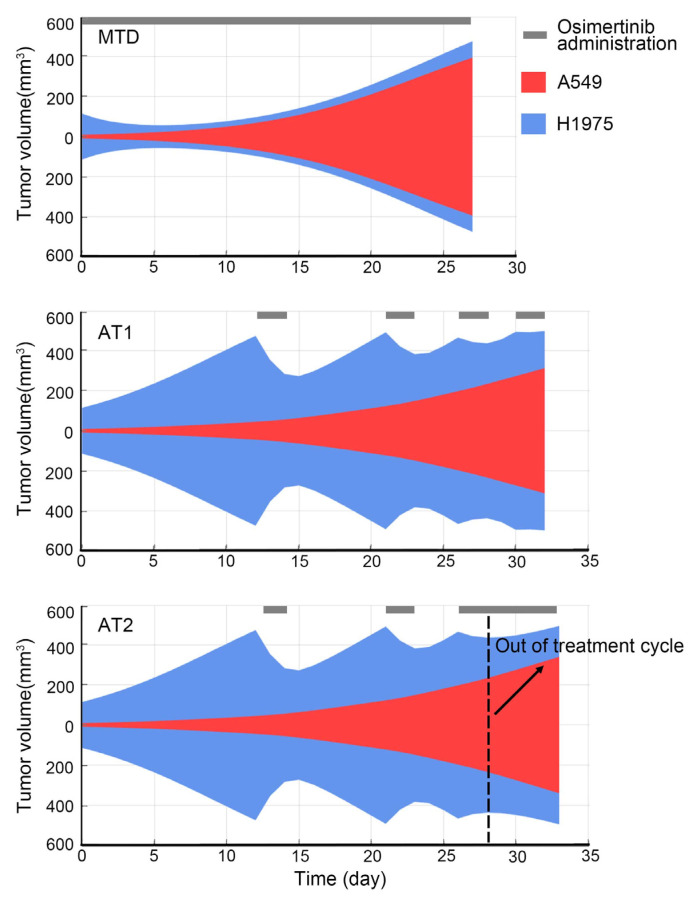
Schematic diagram of subpopulations variation of MTD, AT1, AT2. A general view of H1975 and A549 evolution with time in three therapies. In each subplot of therapy, the blue shade represents the content of sensitive subpopulation H1975, the red shade represents the content of resistant subpopulation A549, the black band represents the osimertinib administration.

To present an overview of each therapy for tumors that mix H1975 and A549, we simulated the subpopulations variation over time in MTD, AT1, and AT2, as shown in (Figure 4). For AT2, because the timing of the switching point was not determined, we interrupted the treatment cycle of AT2 one round earlier than AT1 and switched to high-frequency administration. As shown in (Figure 4, the early termination of the treatment cycle did not reduce the outcome of adaptive therapy. At the later period of AT2, the killing of a sensitive subpopulation can still delay the end event. As for the appropriate time to interrupt the treatment cycle and the suitable case to adopt AT2, it would be explored with a quantitative index.

### 3.5. The Switch Timing of AT2 from Treatment Cycle to High-Frequency Administration

**Figure 5:**
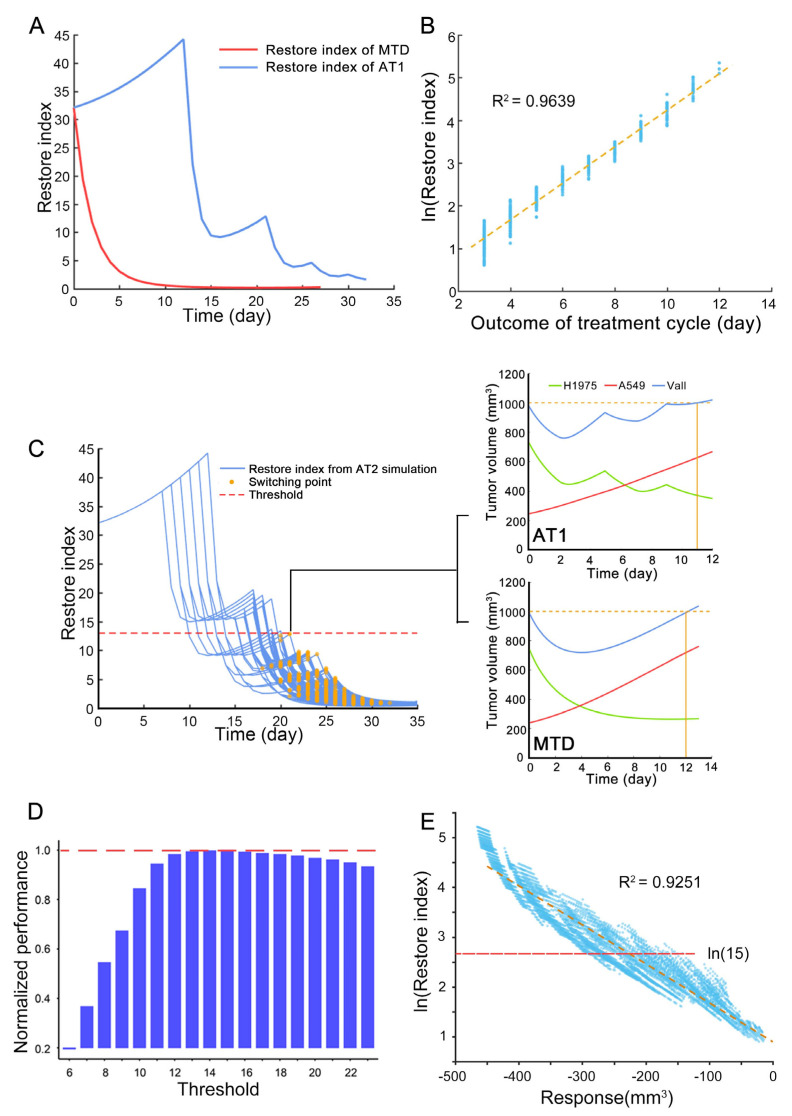
The effect of restore index and the threshold of restore index. (**A**) The change of restore index with therapy time in MTD and AT1. The case was the same with Figure 4. (**B**) The correlation of restore index and duration of treatment cycle with simulated results of the resistant subpopulation with different intrinsic growth rates and carrying capacity. (**C**) The effect of the restore index. The left panel shows the change of restore index over time of filtered AT2 plans, and the switching points indicate the transition to high-frequency administration. The two subplots on the right are the simulation results of AT1 and MTD, and the corresponding tumor component is the restore index at the maximum switching value of the left subplot. (**D**) When AT2 sets different thresholds, the improvement degree of the bad outcome cases of AT1, this improvement degree is normalized with the maximum value. The bad outcome case of AT1 refers to t situation where the outcome of AT1 is worse than MTD. (**E**) The correlation of restore index and tumor volume response for two-days osimertinib administration. The data was from simulation results of the resistant subpopulation with different characteristics.

According to the model, a parameter ‘restore index’ was introduced for better integration the adaptive therapy and MTD. In this system, it is calculated as following equation [5]. The numerator of restore index was the instantaneous reduction rate of H1975 under osimertinib administration, and the denominator was the instantaneous proliferation rate of A549. It would be used to quantitatively evaluate the system reachability because the drug response of H1975 indicated the degree to which the system can be directly regulated, and combined with the proliferation ability of A549 reflected the final effect of the direct regulation of the system. The change of this index versus therapy time was shown in (Figure 5A), where the simulated case of AT1 and MTD was the same as (Figure 4). The changing trend of the restore index was consistent with the composition of the tumor. The setting of this index took into account the drug effect on the tumor and the intrinsic growth rate of resistant cells, so this index can reflect the tumor response of the treatment well. To clarify its ability to predict the outcome of the treatment cycle, we simulated tumors with different aggressiveness resistant subpopulations, whose intrinsic growth rate was defined as 50–200% relative to the typical value of H1975 and carrying capacity was defined as 80–200% relative to the typical value of H1975. And this simulation was adopted with AT1 therapy, the restore index at the start of each treatment cycle and the duration of the corresponding treatment cycle was recorded to analyze the correlation (Figure 5B). Furthermore, this correlation was verified in a public dataset on a prospective trial of intermittent androgen suppression [26]. The model was adopted from the study by West JB et al. [7], and after the model was fitted, the restore index of each patient at the start of treatment cycles 3–5 was calculated. Then the correlation of restore index and duration of treatment cycle was shown in Supplementary Figure S4. The good correlation between the logarithmic restore index and the outcome of the treatment cycle confirmed the effect of this index. Therefore, the restore index was an appropriate indicator for evaluating whether to interrupt the treatment cycle.

(5) $$\mathrm{Restore}\;\mathrm{index}=\frac{E_{OSI}\times \;H\;\times \left(1-\frac{A}{A+H}\right)}{r_{a\;}\times A\times \left(1-\frac{h\times H+A}{K_{a}}\right)}$$

The range of restore index to interrupt the treatment was determined through the first step of estimation the appropriate threshold of restore index for reachability-based adaptive therapy. The plans of AT2 that achieved the same or better outcomes than AT1 were screened out. The restore index changes with therapy time of the above plans were depicted in the left subplot of (Figure 5C). The orange scatters marked the switching points at which each plan interrupted the treatment cycle and shifted to the high-frequency administration. The red dashed line marked the maximum value of switching points in these plans. The simulation of AT1 and MTD was performed with tumor composition of the maximum switching value of the restore index and shown in (Figure 5C) (right subplots). The result showed that at this point, MTD can gain a bit more time to reach the endpoint than AT1. This means that when the restore index was lower than the value indicated by the red dashed line, the treatment cycle of AT1 was meaningless.

According to the second step (see methods part), each integer in 6–23 was used as the threshold of restore index to interrupt the treatment cycle for testing. Finally, the threshold was set to 15, and its selection was based on maximizing the improvement of the poor outcome AT1 case and choosing a larger threshold to minimize the similarity between AT2 and AT1 (Figure 5D). When AT2 was applied to animal experiments for verification, a more direct observable response should be obtained to infer the restore index at that moment. Based on the above simulation data for acquisition of the threshold of restore index, the relationship between the restore index and the tumor volume response for two consecutive days of osimertinib administration was explored, as shown in (Figure 5E). Therefore, when the tumor was adopted the AT2 regimen, if the tumor volume shrank less than 100 mm^3^ after 2-days of osimertinib administration, the treatment would transition to the high-frequency administration.

### 3.6. The Outcome of AT2 Was Not Inferior to AT1 in Tumor System of H1975 and A549

**Figure 6:**
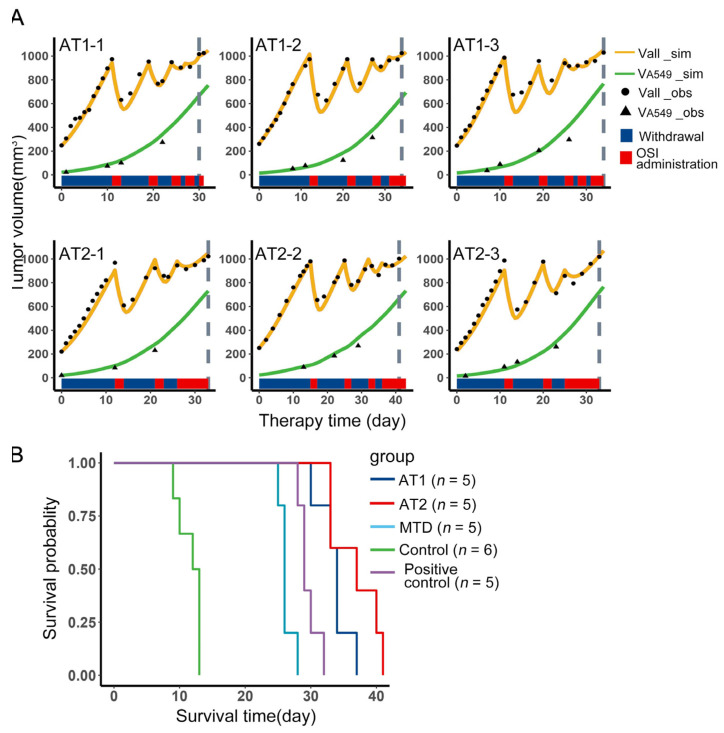
Comparison of therapies in animal experiments. (**A**) Tumor volume versus therapy time in the experiment. The first row contains three samples from the AT1 group, the second row contains three samples from the AT2 group. The black dots were the observations of total volume, and the black triangles were the observations of A549 volume. The yellow curves were the simulation results of total volume, and the green curves were the simulation results of A549 volume. The red and blue color bands below the curves represent the administration and withdrawal of osimertinib, respectively. The gray dashed line indicates the endpoint. (**B**) Survival curves of the Positive control, Control, MTD, AT1, and AT2, the difference between groups is performed by pairwise comparisons use Log-Rank test and shown in Table 1.

Mice inoculated with H1975 and A549 mixed in a ratio of 9:1 were randomly assigned to AT1, AT2, MTD, Control, and Positive control groups. The growth data before grouping was used to calculate the individual parameters, which was applied to the simulation of tumor volume and A549 content versus therapy time. These simulation results could further verify the model. For AT1 and AT2 groups, three samples from each group were presented in (Figure 6A). The black dots were the observations of total volume, and the black triangle were the observations of A549 volume. Both of them were in good agreement with the simulation results. In each subplot, the blue and red color bands represent the treatment at the corresponding therapy time. The number of treatment cycles of AT2 was less than that of AT1. The survival curves of the five groups were shown in (Figure 6B). The following Table 1 showed the corresponding differences between the groups, which was performed by pairwise comparisons using the Log-Rank test. From the results, AT1 and AT2 have achieved better outcomes than MTD and Positive control, and the outcome of AT2 was not inferior to AT1. Therefore, under the AT2 regimen, if the tumor has an aggressive resistant subpopulation, the outcome of AT2 will not be inferior to AT1 and even surpass AT1.

### 3.7. The Suitable Case of AT2

The purpose of reachability-based adaptive therapy was to improve the outcome of AT1 when the tumor has an aggressive resistant subpopulation. To further clarify the applicable fields of AT2, we simulated the outcomes of MTD, AT1, and AT2 in the cases of resistant subpopulation A549 with different values of intrinsic growth rate, competition parameter, and carrying capacity. The change percentage of each parameter was relative to the typical value of the corresponding parameter, and the parameters of sensitive subpopulation H1975 were fixed at the typical value. In addition, the initial volume of H1975 was set to 180 mm^3^, and A549 was set to 20 mm^3^, and the result was shown in Supplementary Figure S5. Figure S5A was shown the time gained by AT1 compared to MTD, when the resistant subpopulation was more aggressive, the time gained by AT1 was less, especially in the upper left corner of Figure S5A. In Figure S5B, the color of the block indicated the better outcome therapy. The outcome of AT1 minus AT2 was shown in Figure S5C, and the yellow and green scatters in the figures represented the sample properties of AT1 and AT2 groups, respectively. AT2 was dominant in the upper left corner area in Figure S5B, which means AT2 was the main choice for cases with aggressive resistant subpopulations. This conclusion was consistent with our optimization purpose. In addition, when the intrinsic growth rate of the two subpopulations declined and approached the growth rate of humans [3], the outcomes of therapies would increase (Figure S5D,E).

## 4. Discussion

It has been studied those factors such as the initial fraction of resistant subpopulations, the proximity of the tumor to carrying capacity, the intrinsic growth rate of a resistant subpopulation, and the rate of cellular turnover affect the time gained of adaptive therapy compared to MTD [7]. These factors can ensure that a sensitive subpopulation has a higher competitive advantage so that intra-competition can inhibit resistant subpopulations for a long time. But when these prerequisites are not met, the advantage of adaptive therapy over MTD will decrease. In this study, we discussed the influencing factors involving intrinsic growth rate, competition intensity, and carrying capacity. We adjusted classic adaptive therapy by interrupting the treatment cycle in advance and switching to high-frequency administration. Since the outcomes of MTD are not inferior to adaptive therapy in these cases, the benefits of killing sensitive subpopulations should be used wisely in adaptive therapy.

In this research, we introduced a new method that considers the benefits of the treatment cycle and high-frequency administration. To find the switching point of the above two treatments, the restore index that takes into account the response of sensitive subpopulation to the drug and the expansion speed of resistant subpopulation is proposed to measure the ability of the drug to regulate the state of the tumor system at the end of each treatment cycle. This is an approximation of the system reachability of this non-linear tumor system. It is found that the restore index can predict the outcome of each treatment cycle through the simulation. Thus, the restore index can evaluate whether to continue the treatment cycle or switch to high-frequency administration.

The switching point plays an important role in the outcome of reachability-based AT. At the switching point, if the treatment cycle continues, there are not enough sensitive cells that can be killed during the high-frequency administration period, then the benefits of MTD cannot be realized. However, if the treatment cycle is interrupted prematurely, the intra-competition cannot be fully utilized, resulting in reduced outcome of reachability-based AT. Therefore, an appropriate switching point can maximize the benefits of both, and the restore index is an effective tool to find this switching point. In addition, the correlation between restore index and the tumor response to the drug was found through the simulation. This correlation converts the calculation of the potential restore index during the therapy into a direct observation of treatment response. Thus, the treatment would transition to the high-frequency administration when tumor volume response of 2-days osimertinib administration was less than 100 mm^3^ in the animal experiment of AT2. In addition, when tumors have more than two subpopulations, they can still be divided into sensitive and resistant subpopulations in terms of drug sensitivity. At this time, the reachability-based adaptive therapy can be further explored for its applicable fields.

When we confirmed the in vivo competition of H1975 and A549, mice inoculated with mixed tumors were given osimertinib for 7 days. Based on the experimental results, the comparison between the observed volume of H1975 and the simulated volume without A549 protection was shown in Supplementary Figure S6. The difference in efficacy indicated that A549 may produce some secretions to support the survival of H1975 during the osimertinib administration, and this phenomenon is also present in other TKIs [27]. This protective effect is enhanced with the increase in A549 content, and intervention in this protective effect is helpful for tumor control. In this study, the difference in competition parameters of H1975 and A549 also indicates that the resources used by the two subpopulations are not the same. Therefore, through the administration of osimertinib and the interference of the distinct metabolic pathway of A549, periodic oscillations of subpopulations can be realized in this tumor system [28], so that system reachability can be maintained for a long time.

## 5. Conclusions

Reachability-based adaptive therapy could improve the performance when the tumor has an aggressive resistant subpopulation; this therapy maximizes the outcome of intra-competition and the killing of sensitive subpopulations by the restore index.

## Figures and Tables

**Table 1:** Comparison of each group.

Experiment Group	AT1	AT2	Control	MTD
AT2	0.1671	/	/	/
Control	0.0035	0.0035	/	/
MTD	0.0035	0.0035	0.0035	/
Positive control	0.0100	0.0035	0.0035	0.0065

## Data Availability

The dataset used during this study is available from the clinical trial by Bruchovsky et al. (http://www.nicholasbruchovsky.com/clinicalResearch.html, accessed on 13 July 2021 or https://github.com/MathOnco/AT_costOfResistance_LVModel/tree/master/data/clinicalData, accessed on 13 July 2021).

## Supplementary Materials

The following are available online at https://www.mdpi.com/article/10.3390/cancers13215262/s1, Figure S1: Cell growth in normal and diluted condition Proliferation conditions of single and mixed cell monitored every 24 h (H1975 in green and A549 in red), the upper is the normal condition, and the lower is the diluted culture medium. Figure S2: H1975 content change. 3D-fluorescent measurement of H1975 for the experiment of in vivo competition. The upper row is the control group, the lower row is the osimertinib group for 7-days osimertinib administration. Figure S3: The effect of osimertinib on H1975 and A549. Figure S4: The correlation of restore index and treatment cycle of public dataset. Figure S5: The suitable case of AT2. Figure S6: Comparison of the observed value and simulated value of the volume change of H1975 in mixed tumors. Table S1: Cell number to volume. Table S2: The content of A549 and H1975 in control and Osimertinib groups at the beginning of the experiment and seven days later. Table S3: Parameter list.
