# Supplementary material for: Optimizing Adaptive Therapy Based on the Reachability to Tumor Resistant Subpopulation
Source: Cancers (Basel). 2021 Oct 20;13(21):5262. doi: 10.3390/cancers13215262 (PMC8582524; doi:10.3390/cancers13215262)
Supplement: Supplementary file 1 [file cancers-13-05262-s001.zip › cancers-1375220-supplementary.pdf]

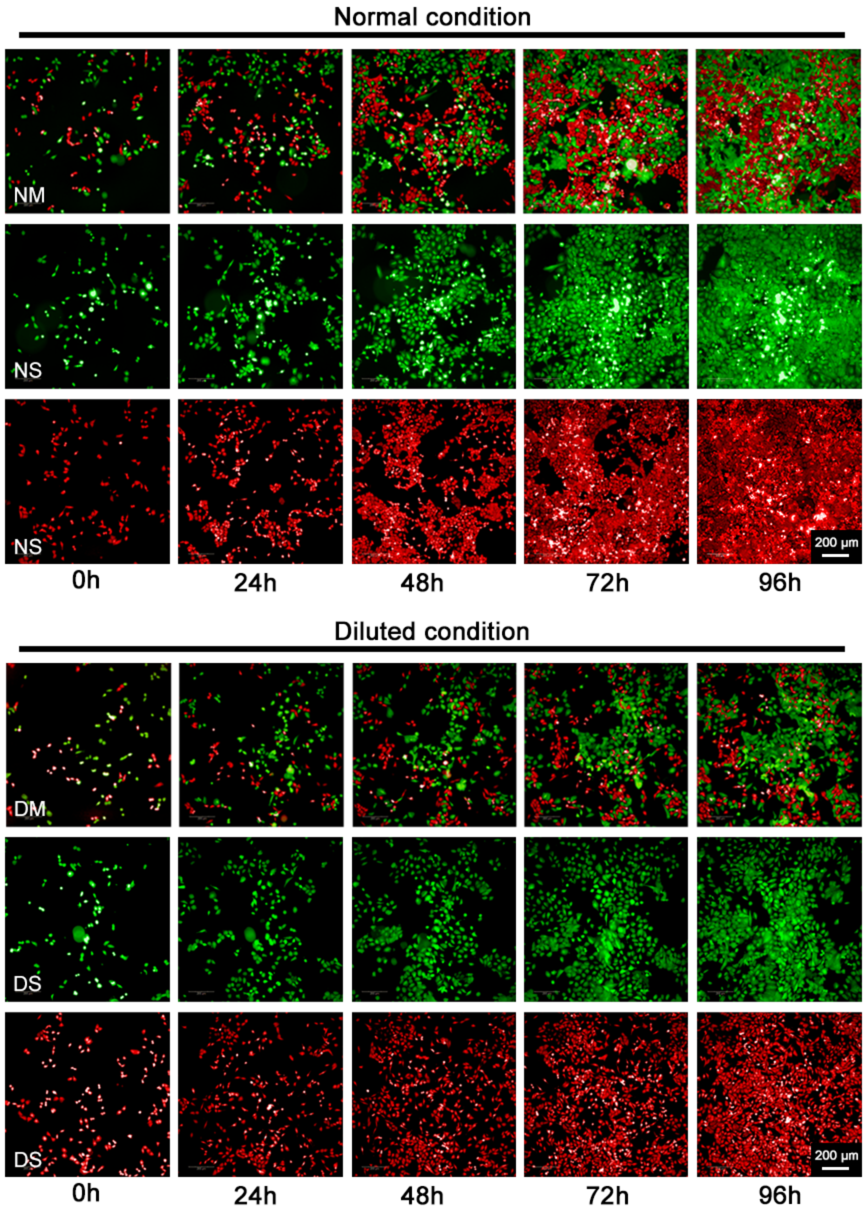

Supplementary Figure S1. Cell growth in normal and diluted condition Proliferation conditions of single and mixed cell monitored every 24 h (H1975 in green and A549 in red), the upper is the normal condition, and the lower is the diluted culture medium.

Supplementary Table S1. Cell number to volume. In order to integrate the observation of tumor volume and the cell number obtained by in vivo imaging, the single cell volume of H1975 and A549 should be calculated and used in subsequent experiments. Linear regression analysis was performed between the cell number of A549 and H1975 measured in the mixed normal tumor and the corresponding tumor total volume to obtain the single cell volume of A549 and H1975. The conversion relationship was shown in following table, and the diagnostic plot of observed values versus predicted values was shown in following figure. This conversion was used in the early period of tumor growth to obtain tumor composition and verified model.

Cell type	Single cell volume(mm^3) (CV)
**A549**	7.274e-07 (29.1)
**H1975**	8.111e-07 (4.9)

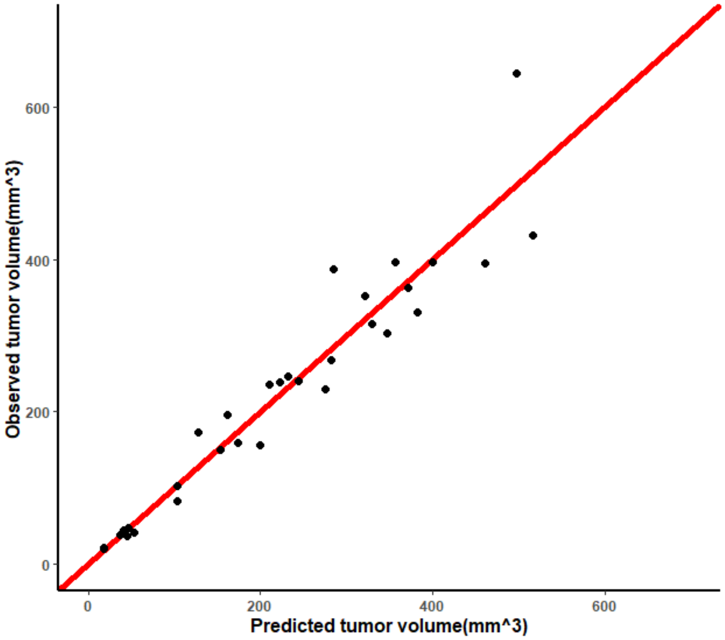

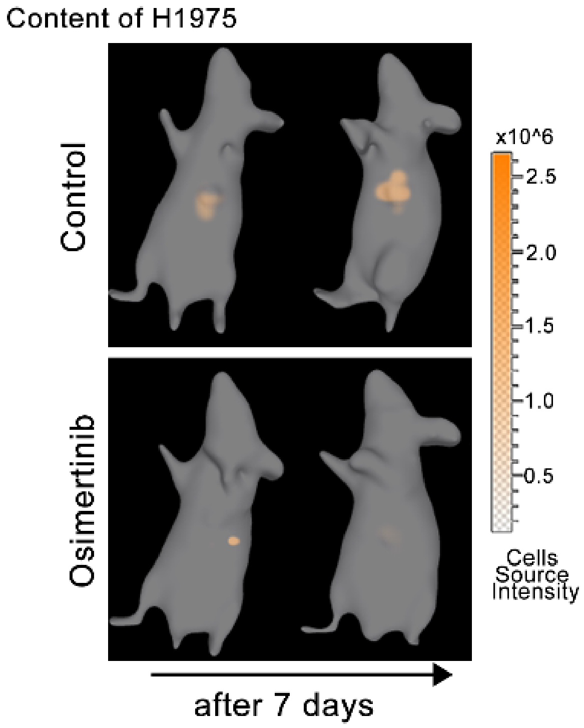

Supplementary Figure S2. H1975 content change. 3D-fluorescent measurement of H1975 for the experiment of in vivo competition. The upper row is the control group, the lower row is the osimertinib group for 7-days osimertinib administration.

Supplementary Table S2. The content of A549 and H1975 in control and Osimertinib groups at the beginning of the experiment and seven days later.

ID	**Content of A549**				**Content of H1975**			
	**Control group**		**Osimertinib group**		**Control group**		**Osimertinib group**	
	Day0	Day7	Day0	Day7	Day0	Day7	Day0	Day7
1	17.68	49.90	17.38	62.48	183.31	454.22	189.80	55.40
2	29.53	70.12	26.33	87.29	256.31	602.65	274.96	77.70
3	21.89	61.90	22.84	98.93	201.96	485.85	202.78	57.18

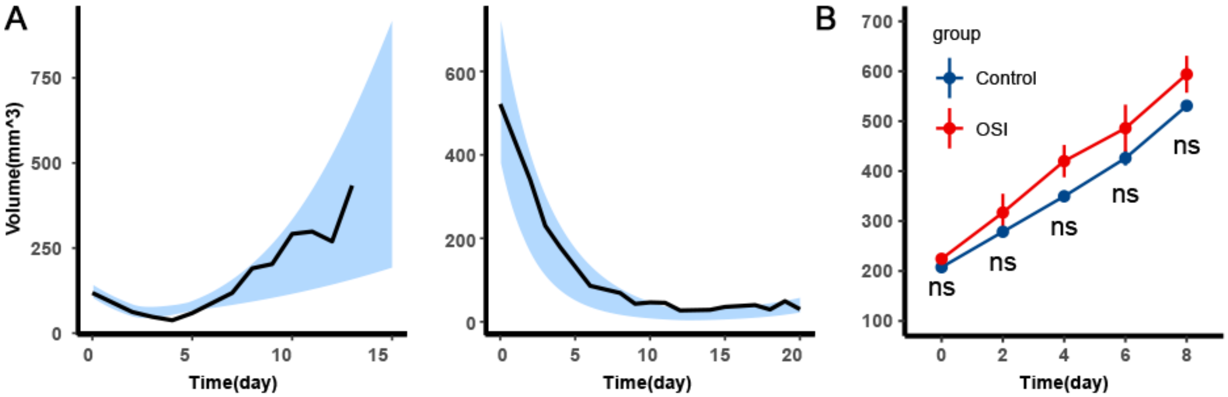

Supplementary Figure S3. The effect of osimertinib on H1975 and A549. (**A**) The effect of osimertinib for H1975. The left side is 2 days of administration from day0, and the right side is 12 days of administration from day0 (*n* = 3 per group). (**B**) Comparing the A549 volume between control and osi group. In osi group, mice inoculated with A549 were given osimertinib for 8 days. The t-test was performed from the initial time point to the end time point, and there was no significant difference between the two groups (*n* = 3).

Supplementary Table S3. Parameter list. The following table is the summarization of model related parameters and estimated by Monolix.

Parameter	Description	Typical Value	Inter-Individual Variation
r_a	A549 intrinsic growth rate	0.184	0.183
r_h	H1975 intrinsic growth rate	0.187	0.254
K_a	carrying capacity of A549	1350	0.316
K_h	carrying capacity of H1975	1410	0.352
a	Competition intensity from A549 to H1975	0.109	2.05
h	Competition intensity from H1975 to A549	0.554	1.56
E_{OSI}	Drug effect of osimertinib to H1975	$1.687 \times r_h + 0.1686$	/
K_{e_OSI}	Metabolic rate of osimertinib	1.31	/

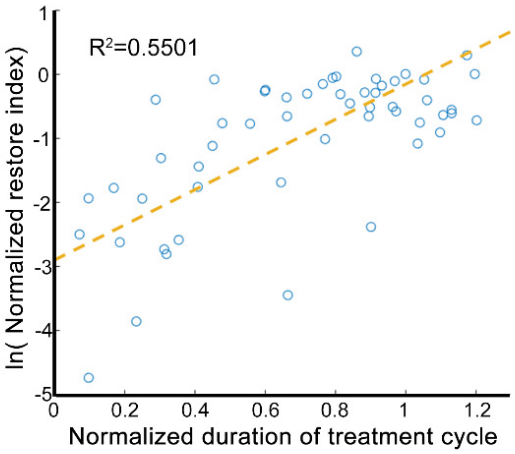

Supplementary Figure S4. The correlation of restore index and treatment cycle of public dataset. After the model fitted, the restore index of each patient at the start of treatment cycle 3–5 was calculated. The restore index and corresponding duration of treatment cycle was normalized to the data of first cycle. Then the correlation of normalized restore index and duration of treatment cycle was analyzed.

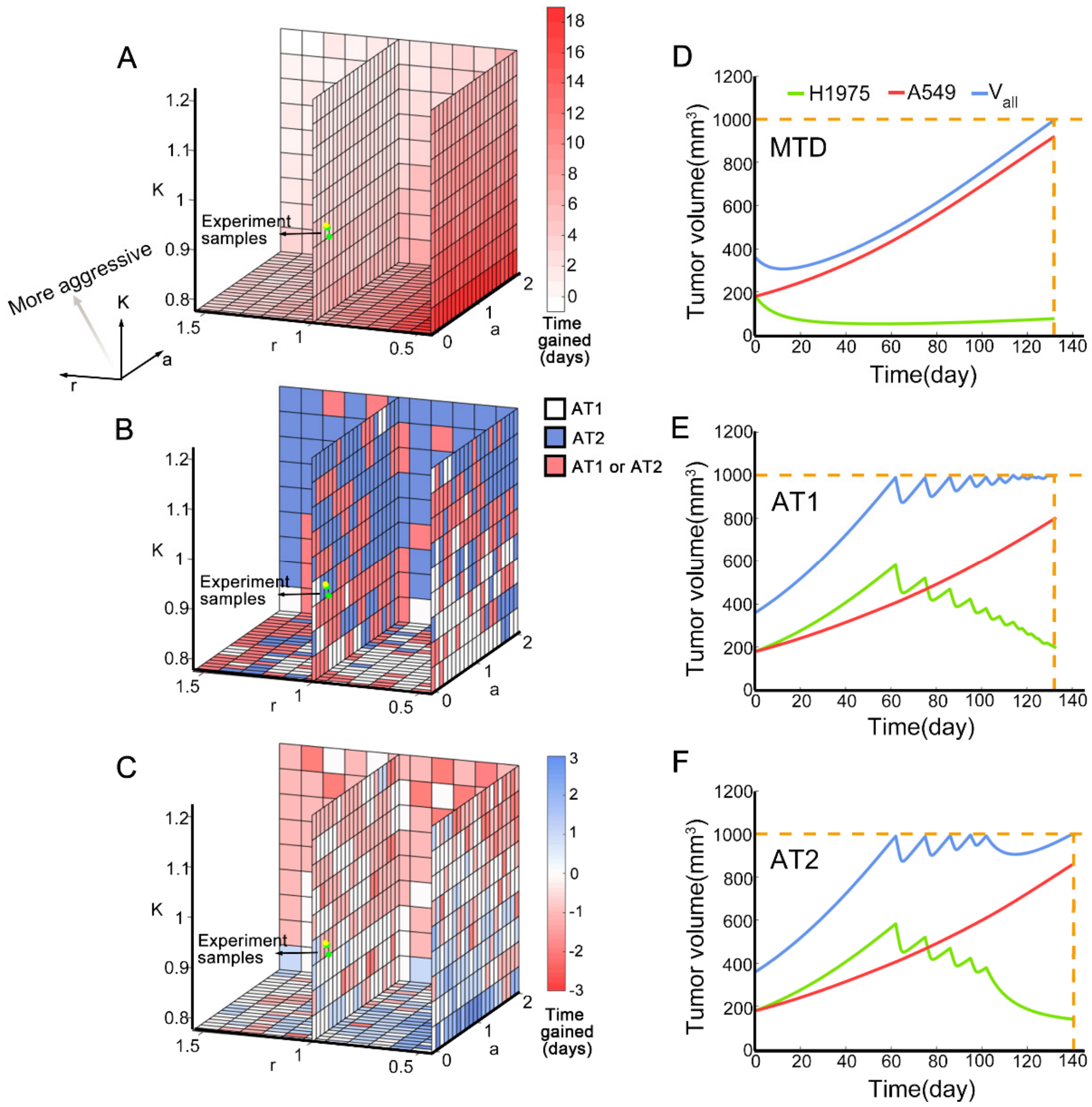

Supplementary Figure S5. The suitable case of AT2. (**A**) A volume slice plan view of outcome of AT1 minus that of MTD, the color blocks indicate the delta value, the yellow scatters indicate the properties of AT1 experiment samples, and the green scatters are AT2 experiment samples. The coordinate in the lower left corner represents the aggressiveness changes of the resistant subpopulation. (**B**) A volume slice plan view of the therapy options for resistant subpopulation of tumors with different biological properties. The color blocks indicate the better therapy, the scatters are same to Figure S5A. (**C**) A volume slice plan view of outcome of AT1 minus that of AT2, the color blocks indicate the delta value, the scatters are same to Figure S5A. (**D**) The simulation result of MTD when intrinsic growth rate of the two subpopulations declined to 10–15% of typical value and the content of resistant subpopulation rose to the same level as that of sensitive subpopulation. (**E**) The simulation result of AT1 therapy of above case. (**F**) The simulation result of AT2 therapy of above case.

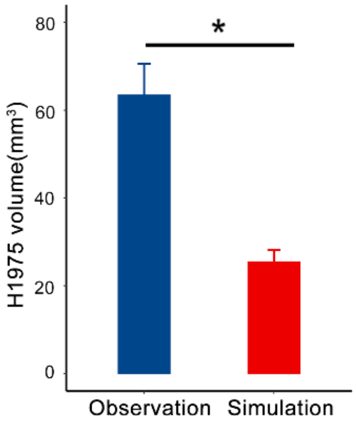

Supplementary Figure S6. Comparison of the observed value and simulated value of the volume change of H1975 in mixed tumors. The observation value was the final volume of H1975 of corresponding A549 outgrowth experiment with 7 days osimertinib administration, and the simulation result was without the support from A549 to the survival of H1975 during osimertinib administration and used the consistent initial value of H1975 and A549 volume, the observation was higher than simulation and the drug effect of osimertinib reduced in mixed tumor significantly (n = 3 per group; paired t-test, p-value = 0.013).
